# Stimuli‐Responsive Crosslinked Chitosan/PVP/PEG Hydrogel Networks for Targeted Drug Delivery and Antimicrobial Performance

**DOI:** 10.1002/ansa.70065

**Published:** 2026-02-10

**Authors:** Danial Mazhar, Sumra Afzal, Safia Hassan, Zahid Imran, Shabana Mehreen

**Affiliations:** ^1^ Department of Chemistry, Islamabad Campus COMSATS University Islamabad Islamabad Pakistan; ^2^ Department of Materials and Environmental Chemistry Stockholm University Stockholm Sweden; ^3^ Catalysis and Sensing Materials Lab, Department of Physics, Islamabad Campus COMSATS University Islamabad Islamabad Pakistan; ^4^ Government College University Lahore Pakistan

**Keywords:** chitosan, ciprofloxacin HCl, diclofenac sodium, drug delivery, hydrogel

## Abstract

In this study, a unique pH‐sensitive PEG/C/PVA hydrogel based on chitosan (C), polyvinyl propylene (PVP) and polyethylenglycol (PEG) crosslinked with tetraethyl orthosilicate (TEOS) was fabricated. This PEG/C/PVA hydrogel was designed to deliver different drugs with distinct release profiles, including diclofenac sodium and ciprofloxacin, and to exhibit antimicrobial activity. As a result of crosslinking with TEOS, the PEG/C/PVA hydrogel exhibits improved swelling characteristics, enabling pH‐dependent drug release. In the characterization study, SEM analysis showed a smooth, porous surface, whereas FTIR confirmed the presence of the amine group of chitosan at 3432.4 cm^−1^, Si─N at 1211.1 cm^−1^ and Si─O─Si at 620.2 cm^−1^, indicating the presence of TEOS. Thermogravimetric analysis (TGA) showed that the synthesized PEG/C/PVA hydrogel exhibits excellent thermal stability. In the swelling study, the maximum swelling (g/g) in water was 25.1, in acidic pH (pH = 2) was 27.57 and at a lower electrolyte concentration, it was 21.3 and 20.6 in NaCl and CaCl_2_, respectively. Based on the release kinetics of diclofenac sodium and ciprofloxacin, we evaluated the performance of the PEG/C/PVA hydrogel and demonstrated good antimicrobial activity. The PEG/C/PVA hydrogel's potential for use in drug delivery systems where precise control over drug release is required is shown by its varied release behaviour. It might be invaluable in situations where drug absorption depends on pH fluctuations or where site‐specific administration is required. This work offers promising results for targeted drug delivery systems by demonstrating the adaptability of a pH‐sensitive chitosan‐based PEG/C/PVA hydrogel crosslinked with TEOS. Subsequent investigations will examine the potential uses and long‐term durability of these PEG/C/PVA hydrogels across diverse biological environments.

## 1 Introduction

There is a consistent need not only for antibiotics and anti‐inflammatory drugs but also for an efficient drug release system, as humans are prone to bacterial infections and subsequent inflammation [1]. There are many side effects of conventional drug delivery systems, such as enzymatic degradation, fluctuations in blood plasma drug levels, decreased concentration at the site of action, pill burden and complex dosage regimens associated with the immediate release and slow absorption of drugs [2, 3]. Recently, there has been a boom in pharmaceutical research on the development of controlled‐release medication systems, as they help maintain plasma drug levels within the therapeutic range and provide the desired therapeutic effect for a more extended period [4]. pH‐sensitive hydrogels have been proposed as an excellent, easy‐to‐synthesize and cost‐effective candidate for the controlled release of drugs [5].

Nowadays, these hydrogels are synthesized by using natural polymers because of their human‐friendly nature and cost‐effective properties [6]. Natural polymers, because of their biocompatibility, biodegradability, softness and superabsorbent absorptivity [7], are chosen for making hydrogels, but due to lower levels of mechanical strength [8]. They are often blended with small amounts of synthetic polymers to enhance the mechanical stability necessary for them to reach the target [9, 10, 11]. Chitosan is one such polymer that is synthesized by deacetylation of naturally existing sugar amine, that is, chitin, which is extracted from crustaceans and fish [12]. Low levels of toxicity [13] and easy degradation by the host enzymes [14] make chitosan a suitable candidate for synthesizing hydrogels for drug delivery uses [15, 16]. The presence of functional groups like ─NH_2_ and ─OH not only makes chitosan hydrophilic but also pH‐responsive [17, 18]. These functional groups also make it chemically reactive to the addition of cross‐linking agents and blenders [19]. As chitosan itself is not stable in an acidic medium [20], so it is cross‐linked with other synthetic polymers to make it stable in acidic media like PEG, PVP, PVA, etc. [21].

Polyethylene glycols (PEG) are biocompatible, hydrophilic, biodegradable, nontoxic synthetic polymers with tunable mechanical properties [22, 23]. In addition to the properties mentioned above, they also have high viscosity and the ability to attach different reactive groups to their terminal sides [23]. So, they are often blended with chitosan to enhance mechanical stability and hydrophilicity. Polyvinyl pyrrolidone (PVP), like PEG, is also a hydrophilic, biocompatible, biodegradable and non‐cytotoxic synthetic polymer [24] which is also added to pure polysaccharide hydrogels to achieve desirable mechanical stability and hydrophilic nature [25].

PEG and PVP are cross‐linked with biopolymers using various crosslinking methods and crosslinkers, such as TEOS, epichlorohydrin, carbodiimide and glutaraldehyde. In all these cross‐linkers, TEOS is non‐toxic, bio‐degradable and bio‐compatible; that's why it is preferred over other cross‐linkers due to their cytotoxicity [26, 27]. These hydrogels are further used for drug delivery applications [28].

Ciprofloxacin HCl is a FDA‐approved antibiotic drug with promising anti‐bacterial activity [29], and its release in the system is dependent on pH changes, which indicate that there should be some pancreatic secretions for its maximal release [30]. Ciprofloxacin HCl is a synthetic chemotherapeutic drug that can be utilized via tablets, capsules and injections, but the maximum bioavailability of the drug is 52% when it is delivered through oral administration method [31].

Diclofenac sodium is an FDA‐approved first non‐steroidal anti‐inflammatory drug (NSAID), which is used to treat rheumatoid arthritis and osteoarthritis [32]. Diclofenac sodium is a non‐selective drug that can be administered orally, rectally, transdermally or intramuscularly. Although the oral route has many side effects, such as gastrointestinal bleeding and central nervous system complications [33]. But oral administration methods are still preferred, as absorption by this route is rapid [31]. Due to low skin permeability and uncontrolled release of diclofenac sodium [33]. The hydrogels of different biopolymers, such as chitosan, alginate, guar gum, acrylic acid and their derivatives, are being used for controlled release of drugs such as ciprofloxacin HCl and diclofenac sodium because of their modification to be responsive to different pH or external conditions [34].

Drug delivery applications have shown interest in pH‐responsive chitosan‐based PEG/C/PVA hydrogels, including PEG and PVA (polyvinyl alcohol). These materials are appealing for regulated drug delivery due to their stimulus‐responsive properties, biocompatibility and biodegradability. Nonetheless, there are problems and research gaps that need further investigation across different pH ranges. It is essential to know and regulate the pH range at which the PEG/C/PVA hydrogel swells or deswells. The primary goal of this research project was to develop a novel pH‐responsive chitosan‐based PEG/C/PVP PEG/C/PVA hydrogel. Further, we have studied the swelling response at different pH levels and electrolyte concentrations and in an aqueous medium at various time intervals. Swelling studies were performed in multiple media, including deionized water, buffers and salt solutions. Both drugs were loaded into separate PEG/C/PVA hydrogels, and their drug release profiles were analysed in simulated gastric fluid (SGF) and simulated intestinal fluid (SIF) using UV‐visible spectrometry.

## 2 Materials and Methods

### 2.1 Chemicals and Reagents

Chitosan (C) powder (MW = 90000 g/mol; DDA >75%, density = 0.15–0.30 g/cm^3^), PVP (MW = 111.144 g/mol, CAS NO. 9003‐39‐8, density = 1.2 g/cm^3^), TEOS MW = 208.33 g/mol, hydrochloric acid (HCl), sodium hydroxide (NaOH), acetic acid (CH_3_COOH), calcium chloride (CaCl_2_), sodium chloride (NaCl), ethanol (C_2_H_5_OH) MW = 40.069 g/mol with 99.8% purity were acquired from Sigma Aldrich. Deionized water was used throughout the experimentation.

### 2.2 Synthesis of PEG/C/PVA Hydrogel

For the synthesis of the PEG/C/PVA hydrogel, the solution‐casting method was used. Chitosan (0.8 g) was dissolved in 2% acetic acid (100 mL). PEG (0.1 g) and PVP (0.1 g) were separately dissolved in 20 mL of deionized water. These two solutions were added to the chitosan solution after 1 h, with constant stirring at 80°C. TEOS (20 µL) was mixed with 10 mL of ethanol, which was then added dropwise to the blend solution. The blend solution was kept stirring for an additional 5–6 h at the same temperature. After this blend was poured into the petri dish, it was allowed to dry for 3–4 days at room temperature. To prevent moisture adsorption, the dried PEG/C/PVA hydrogels were removed from Petri dishes and placed in a desiccator. Mechanism 1 illustrates the proposed interactions between the PEG/C/PVA hydrogel's constituents.

### 2.3 Drug‐Loaded PEG/C/PVA Hydrogel

Chitosan (0.8 g) was dissolved in 2% acetic acid solution (100 mL). PEG (0.1 g) and PVP (0.1 g) were separately dissolved in 20 mL of deionized water. These two solutions were added to the chitosan solution after 1 h, with constant stirring at 80°C. TEOS (20 µL) solution was prepared in 10 mL of ethanol and added dropwise to the blend solution. Then, 20 mL of solvent (distilled water) was used to dissolve 50 mg of the model drug, which was then added to the blend solution. The mixture was stirred for an additional 5–6 h at the same temperature. The mixture was then added to a petri dish and left to dry for 3–4 days at ambient temperature. To prevent moisture adsorption, the dried PEG/C/PVA hydrogels were removed from Petri dishes and placed in a desiccator.

### 2.4 Preparation of SGF and SIF and Drug Release Study

The published method [35] was used to prepare intestinal fluid (pH = 6.8) and gastric fluid (pH = 1.2). To make SGF (pH 1.2), 1 g of NaCl was dissolved in water, then 3.5 mL of strong HCl was added and vigorously mixed to set the pH to 1.2. SIF (pH 6.8) is prepared by mixing 250 mL of 0.2 M KH_2_PO_4_ with 118 mL of 0.2 M NaOH. Thoroughly mix until the final pH is 6.8.

For the drug release investigation, 100 mL of SGF (pH 1.2) was poured into a 500 mL beaker. The hydrogel sample was submerged in the solution and kept at 37°C for 180 min. To maintain a consistent volume, a 5 mL aliquot was taken every 15 min and replaced with an equal volume of fresh SGF. A UV‐visible spectrophotometer at 264 nm was used to quantify the amount of drug released. The exact process was performed with SIF (pH 6.8) to assess drug release behaviour under intestinal circumstances. Reference drug solutions were produced in both SGF and SIF. The release patterns were plotted, and the quantity of released drug was calculated using a previously developed absorbance‐concentration calibration curve.

## 3 Characterizations

### 3.1 Swelling Experiments

#### 3.1.1 Swelling Index in Distilled Water

To assess swelling response in deionized water, a small piece of PEG/C/PVA hydrogel was cut, weighed and immersed in deionized water. After a specific time interval, the water was removed, and excess water was wiped out with filter paper. The weight of the swollen PEG/C/PVA hydrogel was determined. The swelling ratio was determined by the following Equation (1):

(1) $$\mathrm{Swelling}\;\;\left(\frac{g}{g}\right)=\frac{W_{s}-W_{d}}{W_{d}}\;$$

where *W_s_* is the weight of the swollen PEG/C/PVA hydrogel, and *W_d_* is the weight of the dry PEG/C/PVA hydrogel.

The swelling behaviour was examined at different pH levels (2, 4, 7, 8, 10 and 12) in non‐buffered solutions (aqueous solutions adjusted to 2, 4, 7, 8, 10 and 12 pH using 0.1 M HCl and NaOH). Pre‐weight samples of PEG/C/PVA hydrogels were taken, immersed in non‐buffer solutions one by one, and left for 2 h. After that, the solutions were removed, and filter paper was used to collect the excess solutions. The weight of PEG/C/PVA hydrogel was also determined, and the swelling ratio was calculated by Equation (1). Solutions of various concentrations (0.1, 0.2, 0.4, 0.6, 0.8 and 1.0 mol/L) of NaCl and CaCl_2_ were prepared to check the swelling response in electrolyte solutions. The dried PEG/C/PVA hydrogel was cut into small pieces and weighed before dipping into the solutions. After 2 h, the salt solutions were eradicated, and the weight of the PEG/C/PVA hydrogel was determined. The swelling ratio was calculated from Equation (1).

To determine the gel content, a piece of dried PEG/C/PVA hydrogel was taken, weighed and transferred to a stainless‐steel cloth. After boiling the sample in a Soxhlet extractor for 8 h, it was removed and placed in a vacuum at 60°C for 60 min. The sample weight was measured every 15 min until it stabilised. The gel fraction was determined by using the given Equation (2):

(2) $$\mathrm{Gel}\;\mathrm{fraction}\;\left(\%\right)=\frac{W_{g}}{W_{o}}\;\times 100$$

where *W_g_* is the weight of the sample removed in grams and *W_o_* is the initial weight of the sample in grams.

### 3.2 Fourier Transform Infrared Spectroscopy

The PEG/C/PVA hydrogel sample was analysed at a scan rate of 200 scans to obtain a high‐signal‐t0‐noise spectrum at resolution of 4.0 cm^−1^. The spectrum was obtained in the 4000–400 cm^−1^ frequency range in transmittance mode to confirm the physical and chemical interactions in the synthesized PEG/C/PVA hydrogel using a Nicolet 6700 FTIR spectrophotometer (USA).

### 3.3 Thermogravimetric Analysis

A Shimadzu DTG‐60H analyser (detector serial number C30574300132TK) was used to determine the thermal stability of the synthesized PEG/C/PVA hydrogel. The sample was tested over the temperature range of 25–600°C with a ramp rate of 10°C min^−1^. The samples were analysed in a nitrogen atmosphere at a flow rate of 15 mL/min.

### 3.4 Scanning Electron Microscopy

To confirm the morphology, the synthesized PEG/C/PVA hydrogels were examined under scanning electron microscopy (SEM) using MIRA3 XMU TESCAN (SEM HV: 10.00 kV). Various sputtering coating was also applied to modify the magnification of SEM images.

### 3.5 In Vitro Antimicrobial Analysis

The antibacterial analysis of PEG/C/PVA hydrogels was performed using the Kirby–Bauer Disc Diffusion method [36], against the bacterial strains *Escherichia coli*, *Pseudomonas aeruginosa*, *Acinetobacter calcoaceticus*, *Salmonella typhi*, methicillin‐resistant *Staphylococcus aureus* and *Klebsiella pneumoniae*. In this method, bacteria were inoculated on Muller–Hinton agar. Discs of about 9 mm in diameter were cut off from the PEG/C/PVA hydrogels. The same‐sized discs were also prepared from Whatman filter paper to load the precursors (PEG, PVP and TEOS) and the reference ciprofloxacin. The precursors were loaded on the disc; prepared disks were placed on agar having lawns of the respective bacteria and incubated at 37°C overnight.

### 3.6 Degradation

Hydrogel degradation was studied in PBS (pH 7.4) at 37°C for 7 days. Initially, the dry hydrogel samples (45 mg each) were weighed, divided into three sets and submerged in PBS. The samples were taken at predefined time intervals (1, 3, 5 and 7 days), blotted to remove moisture, oven‐dried and weighed. After weighing, the samples were reintroduced to the PBS solution. This procedure was repeated with time intervals, blotted, oven‐dried and weighed before being reintroduced to the PBS solution. This process was repeated at each time interval to get precise degradation rates. Equation (2) was used to calculate the degradation percentage,

$$\mathrm{Degradation}\;\left(\%\right)=\frac{W_{i}-W_{t}}{W_{t}}\;\times 100$$

where *W_i_* denotes the initial weight of the hydrogels and *W_t_* denotes the weight at time *t*.

### 3.7 Mechanical Strength Analysis

Tensile specimens were cut from the middle parts of the produced PEG/C/PVA hydrogels with a diameter of 1.0 mm using scissors. Tensile strength and total elongation at break were measured at room temperature with a CMT 5105 PC‐controlled mechanical testing equipment (MTS Co. Ltd., USA) at a constant crosshead speed of 5 × 10^−3^ mm/s for different temperatures. To ensure data accuracy, three parallel tests were performed for each wire diameter.

## 4 Results and Discussion

### 4.1 Functional Group Analysis

**FIGURE 1:**
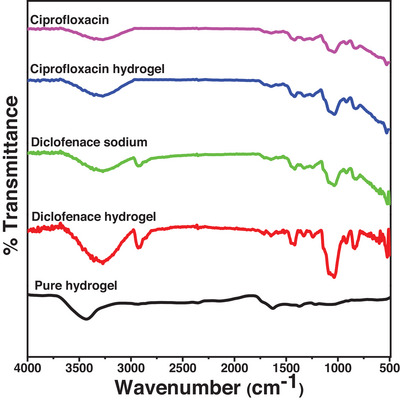
FTIR spectrum of PEG/C/PVP hydrogel.

The FT‐IR spectra of the synthesized pure PEG/C/PVA, pure drug samples and drug‐loaded hydrogels are shown in Figure 1. The pure PEG/C/PVA spectrum shows a broad absorption band at 3432.54 cm^−1^, which is due to the ─NH group of chitosan [37], also masked by O─H stretching vibrations of inter and intramolecular hydrogen bonding [38]. The band at 2930.93 cm^−1^ corresponds to the characteristic C─H stretching vibration [39]. The hydrogen bonding is confirmed between the constituents of the PEG/C/PVA hydrogel by the absorption band at 1628.16 cm^−1^. This hydrogen bonding may be present between the carbonyl group of PVA and the hydroxyl groups of chitosan and PEG, as shown in Figure 1. The band at 1628.16 cm^−1^ can also be assigned to the C═O stretching of the acetamide group of chitosan [40]. The band at 1211.11 cm^−1^ is assigned to the new Si─N bond formed by the interaction between TEOS and chitosan. The absorption bands at 1089.76 and 1375.34 cm^−1^ are associated with the ─C─O─C cyclic and acyclic linkages, respectively. The presence of the siloxane bond (Si─O─) is masked in the absorption band at 1089.76 cm^−1^ due to TEOS. The gel content of the synthesized PEG/C/PVA hydrogel was 89.65%, confirming the formation of a cross‐linked network by TEOS. The absorption band at 620.22 cm^−1^ corresponds to the Si─O─Si linkages of TEOS, which is responsible for cross‐linking between chitosan, PVP and PEG [41]. The FTIR spectra of diclofenac sodium are shown in the figure. COOH stretching vibrations in diclofenac sodium show a peak at 3277 cm^−1^, while C═C and N─H stretching vibrations have infrared bands at 1640 and 3385 cm^−1^, respectively. The findings are closely similar to the prior study.

Compared to the spectra of pure drugs, diclofenac sodium exhibits strong bands near 1570–1580 cm^−1^(carboxylate/aryl C═C or COO^−^ stretching) and a diagnostic C─Cl stretch at about 746 cm^−1^ [42, 43], while ciprofloxacin HCl shows characteristic absorptions at 3525–3385 cm^−1^ (O─H / N─H), 1700–1625 (cm‐1quinolone C═O/aromatic C═C) and fingerprint bands in the 1100–800 cm^−1^ region related to C─F and aromatic ring vibrations [44]. These observations agreed with previously published FT‐IR assignments for ciprofloxacin and diclofenac. The broadening and reduction of intensity of the OH/NH band at about 3433 cm^−1^ after drug loading indicated hydrogen bonding between the ─OH/─NH groups of hydrogel and functional groups of the drug molecules, while the 1628 cm^−1^ band shows a slight shift or decreased intensity, which indicates the interaction between the drug molecules and C═O and amide groups of chitosan/PVA. This can be taken as an indication of good incorporation and interaction of the drugs within the PEG/C/PVA hydrogel matrix, as evidenced by reduced intensities or slight shifts in the drug peaks at 1570 cm^−1^ (COO^−^) for diclofenac and 1700–1625 cm^−1^ (C═O) for ciprofloxacin.

### 4.2 Thermogravimetric Analysis

**FIGURE 2:**
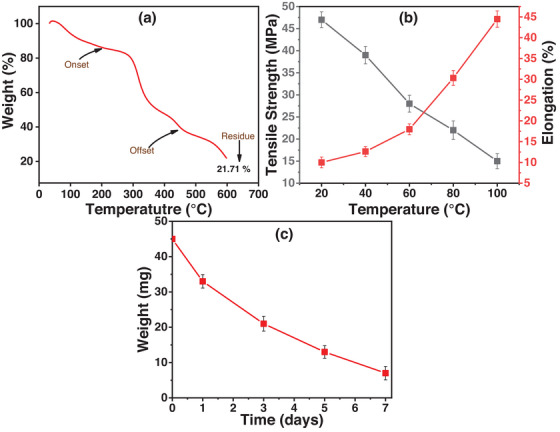
(a) TGA thermogram of synthesized hydrogel. (b) Tensile strength and elongation at break of hydrogel with changing temperature. (c) Degradation of hydrogel in PBS solution.

The thermal stability of the PEG/C/PVA hydrogel sample was studied by thermogravimetric analysis. Figure 2a shows the thermogram plotted as a function of weight (%) and temperature (°C). The thermogram in the figure exhibits that degradation occurs in three steps. The weight loss in the first step, between 43.09°C and 250.89°C, was due to the elimination of water and moisture from the sample, and the total weight loss was 17.26%. In the second step, between 256.31°C and 389.26°C, 35.77% weight loss was due to the dissociation of hydrogen bonds and secondary interactions between the polymer chains of chitosan, PEG, PVP and TEOS. While in the third step, between 397.40°C and 600°C, the backbone of polymer chains was ruptured, and the PEG/C/PVA hydrogel network was degraded into small fragments [45]. According to the literature, freezing‐bound water, non‐freezing water and free water are the types of absorbed water present in the hydrophilic polymer [46]. At temperatures up to 100°C, free water is released because it does not interact through secondary forces. Non‐freezing water forms H‐bonds with polymer chains, whereas freezing water forms weak forces of attraction with the polymer. Thus, above 100°C, these two forms of water are released from the polymer chains [47]. Moreover, the hydrogen bonding between PEG, PVP and other ingredients, as well as covalent interactions between chitosan and TEOS, are responsible for enhanced thermal stability [48].

### 4.3 Morphological Studies

**FIGURE 3:**
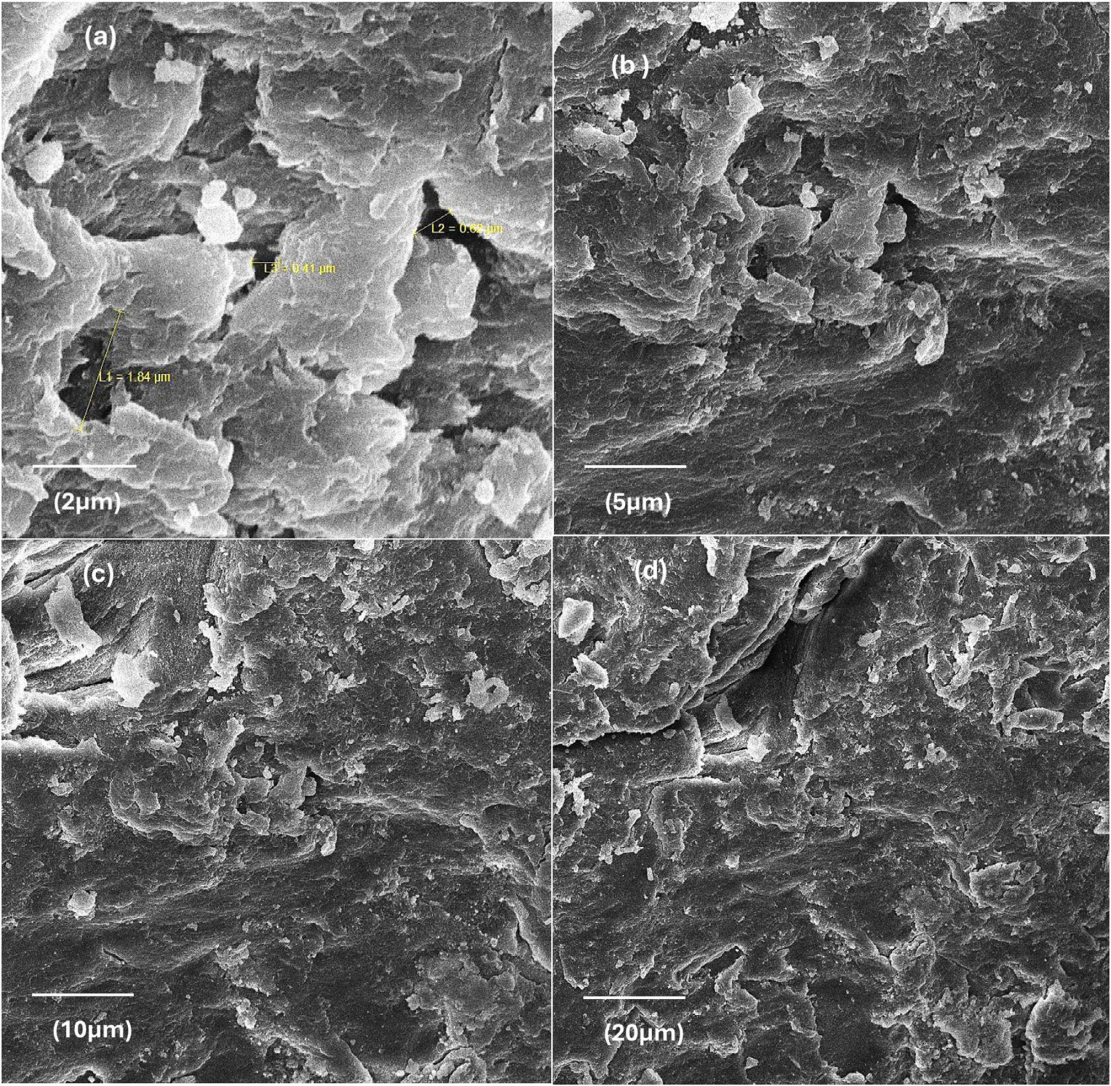
SEM images of PEG/C/PVP hydrogel at different resolutions.

**FIGURE 4:**
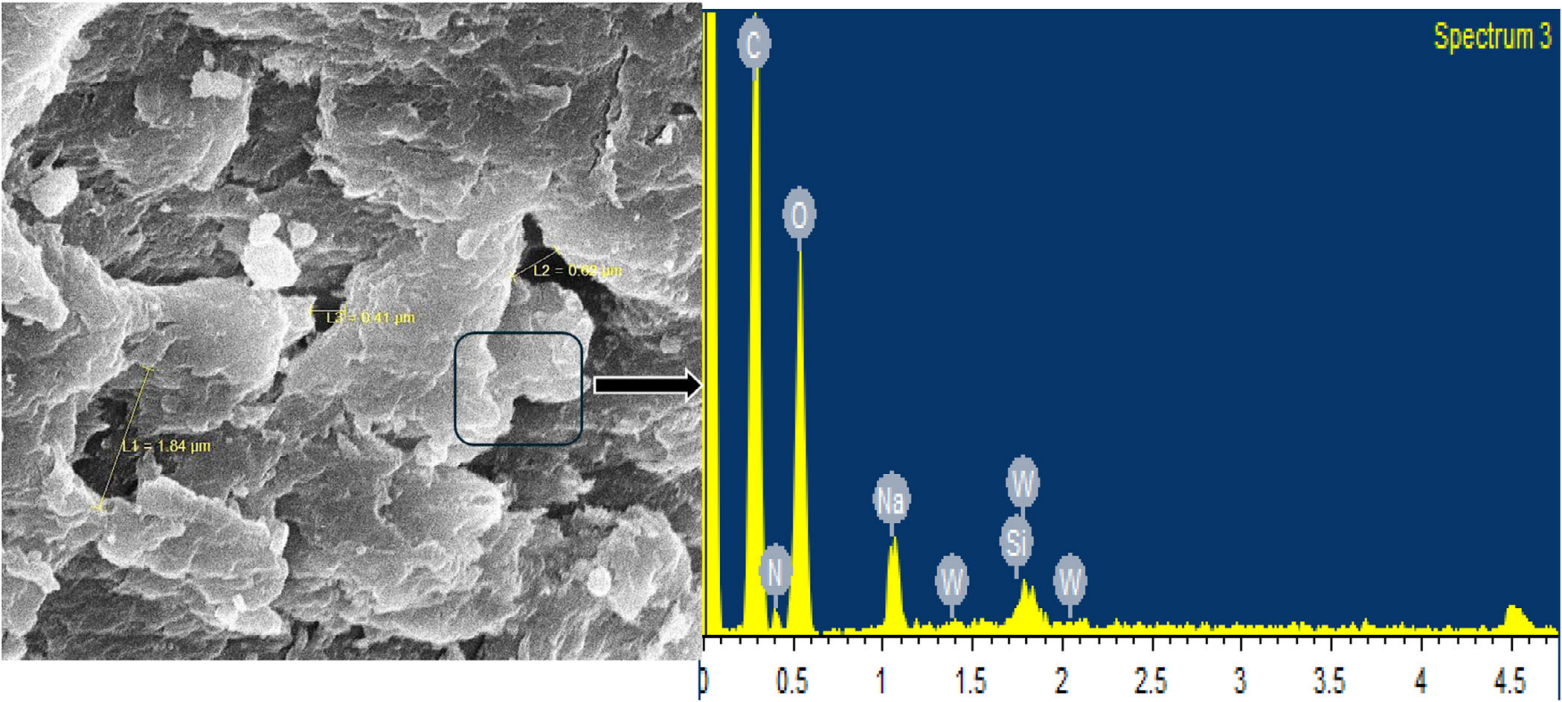
SEM with EDX of PEG/C/PVA hydrogel.

**FIGURE 5:**
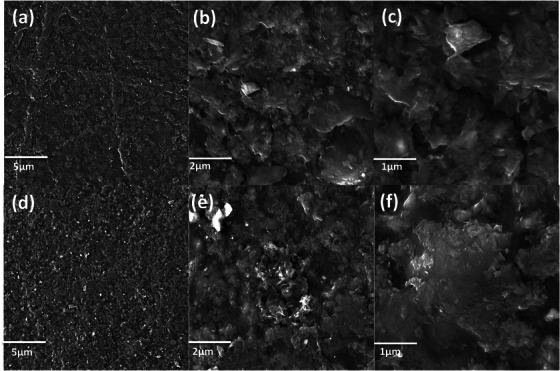
SEM images of diclofenac Na‐loaded PEG/C/PVP hydrogel (a,b,c) and ciprofloxacin HCl‐loaded PEG/C/PVP (d,e,f) at different resolutions.

The surface morphology of the PEG/C/PVA hydrogel and the cross‐linking among the polymers were confirmed by SEM. The micrographs of the synthesized PEG/C/PVA hydrogel at different resolutions are shown in Figure 3, and SEM with EDX is demonstrated in Figure 4, which exhibits the ruptured morphology and porous structure of PEG/C/PVA hydrogel which is responsible for swelling [49]. All the samples appeared transparent microscopically. Figure 4 shows an EDX spectrum of PEG/C/PVA hydrogel crosslinked with TEOS. The presence of C, O and N validates the organic polymeric matrix, while the appearance of Si in the spectrum suggests that TEOS was successfully included as a crosslinking agent. The minor Na peak observed in the EDX spectrum may originate from trace impurities in the starting materials or from handling‐related contamination, since NaOH was not directly used during synthesis. Whereas the SEM sample holder or an instrument artefact most likely causes W peaks. Figure 5 shows the SEM images of both diclofenac Na‐loaded and ciprofloxacin HCl‐loaded PEG/C/PVP hydrogels, which show a similar porous structure to that of the unloaded hydrogels.

### 4.4 Mechanical Properties of Hydrogel

Mechanical parameters of the hydrogel at different temperatures, such as tensile strength and elongation at break, are reflected in Figure 2b. The tensile strength of the hydrogel decreases with the rise of temperature. In contrast, its elongation at break significantly increases. This suggests that at high temperatures, the thermal mobility of polymer chains increases, destroying the ordered structure of the PEG/C/PVA molecular network. As a result, the hydrogel becomes much more flexible yet mechanically weaker with increasing temperature.

### 4.5 Degradation Analysis

Biodegradation is defined as the natural process of breakdown of biomaterials. In clinical applications, however, it describes those biological processes that progressively degrade an implanted material in the body. Knowledge of degradation behaviour is essential because the residence time of a biomaterial determines its ability to execute a required medical function. Polymeric hydrogels are especially useful in this regard, as their structure can be readily modified to provide for controlled drug release [50]. Degradation of the PEG/C/PVA hydrogel was studied at 37°C for 7 days in PBS (pH 7.4). As shown in Figure 2c, the hydrogel exhibited continuous weight loss, indicating progressive degradation under physiological conditions. A rapid initial weight loss was observed on Day 1 due to surface erosion and the dissolution of loosely bound polymer chains. Subsequently, the hydrogels exhibit a steady and progressive decrease in weight from Day 1 to Day 7, reflecting the hydrolytic breakdown of the internal polymeric network. The degradation could result from weak interactions, such as van der Waals and hydrogen bonding, or from strong interactions, such as glycosidic and covalent bonding. These interactions weakened due to the degrading nature of hydrogels in PBS at 37°C and pH 7.4.

### 4.6 Swelling Analysis

Swelling behaviour in hydrogels significantly impacts their physical structure, interactions with solvents and solutes and, consequently, permeability and solute rejection performance. The swelling study is primarily intended to assess the hydrogel's stability. This multidimensional process is determined by parameters such as solvent–polymer affinity, the existence of osmotically active species (e.g., mobile counter‐ions), and the degree of polymer cross‐linking. The equilibrium water content, or swelling capacity, of hydrogels is significant in drug delivery applications, where water uptake governs the diffusion and release of the encapsulated drug.

**FIGURE 6:**
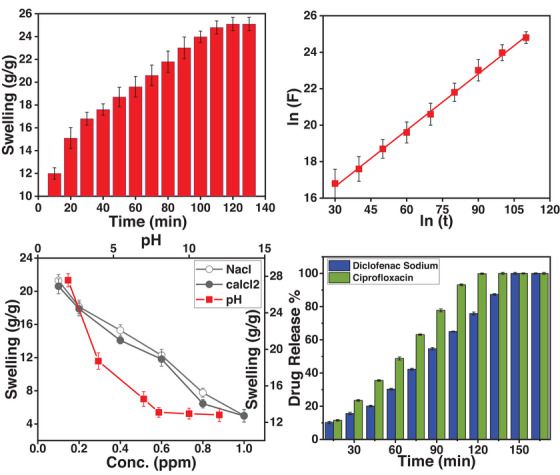
Time dependent swelling in deionized water (a), ln (t) plotted against ln (F) (b), swelling at different pH and different concentration of electrolytes (c), and drug release profile in SIF(d).

The drug release mechanism in PEG/C/PVA hydrogel mainly depends on its ability to absorb water; therefore, assessing the swelling behaviour is essential for understanding the release kinetics [51]. The swelling behaviour depends upon hydrophilicity, hydrophobicity, cross‐linking density, osmotic pressure and pH of PEG/C/PVA hydrogel [52]. The swelling of the PEG/C/PVA hydrogel in deionized water is shown in Figure 6a as a function of time, indicating that swelling initially increases linearly and attains equilibrium within 120 min. First, water diffuses into the PEG/C/PVA hydrogel network through interactions between hydrophobic and polar groups, and the network expands as the polymer chains swell upon hydration. An additional absorption of water occurs due to osmotic pressure, which is opposed by the elastic retractive forces of the cross‐links, as shown in *S.1*. At the equilibrium, a balance is maintained between the osmotic pressure and the elastic retractive forces of the polymers [53]. After 120 min, the equilibrium point is reached due to a decrease in the polymers' hydrophilicity, as their hydroxyl groups are fully occupied. The maximum swelling observed in 120 min is 25.1(g/g).

When the water molecules invade the PEG/C/PVA hydrogel's surface, the diffusion mechanism arises, which can be Fickian or non‐Fickian. Ritger–Peppas equation [54] was used to explain the diffusion phenomenon,

(3) $$F=Kt^{n}\;$$

where ‘*F*’ is the fractional swelling at time ‘*t*’ (min), ‘*k*’ is the swelling rate constant, and ‘*n*’ is the swelling exponent. The values of diffusion parameters such as ‘*k*’ and ‘*n*’ were calculated from PEG/C/PVA hydrogel swelling data, which is plotted between ‘*ln t*’ and ‘*ln F*’ as shown in Figure 6b. As the value of ‘*n*’ is less than 0.5, it means that diffusion is Fickian according to Fick's law. Different diffusion parameters were also calculated and are shown in Table 1. The gel fraction of the hydrogel was determined to be 89.65%, indicating the production of a highly crosslinked and stable polymeric network. A high gel content suggests that most polymer chains are successfully incorporated into the three‐dimensional structure, thereby reducing sol fraction loss. This strong crosslinked structure increases the gel strength and mechanical stability of the hydrogel. The findings suggest that TEOS served as an effective crosslinking agent, facilitating strong interlinking among chitosan, PEG and PVA chains and yielding a robust hydrogel matrix.

**TABLE 1:** Diffusion parameters of synthesized hydrogel.

Parameters	Hydrogel
*n*	0.1318
Intercept	13.53689 ± 0.13217
Slope	0.10327 ± 0.00177
Regression (*R* %)	0.99794
Gel fraction (%)	89.65

The swelling behaviour of PEG/C/PVA hydrogel is also studied at different solution pH (2–12), as shown in Figure 6c. It exhibits pronounced swelling at acidic pH (pH = 2) as compared to neutral and alkaline conditions, mainly due to the functional group of chitosan and PEG, as PVP is not significantly affected by the pH [55]. The reason for maximum swelling at acidic pH is the protonation of the chitosan amine group, which is ionized to NH_3_^+^. The NH_3_^+^─NH_3_^+^ repulsion increases osmotic pressure, leading to the breakdown of secondary interactions, such as hydrogen bonding, which allows additional water to be absorbed into the PEG/C/PVA hydrogel network [56, 57]. Deprotonation of the amine functional group occurs as the pH increases in neutral and alkaline conditions, as the pKa value of the amine group is close to 6.5 [58], in which NH_3_^+^ groups convert back to NH_2_ moieties. It promotes the formation of secondary attractions between polymer chains, thereby decreasing the swelling of the PEG/C/PVA hydrogel in both basic and neutral media [59].

The swelling behaviour of PEG/C/PVA hydrogel also depends upon the ionic concentration in the external medium [39]. Figure 6c shows the swelling behaviour of the PEG/C/PVA hydrogel in NaCl and CaCl_2_, respectively, revealing that swelling decreases with increasing salt concentration. The ionic interactions between the mobile and fixed ions generate osmotic pressure between the external solution and the PEG/C/PVA hydrogel, which can be used to determine the swelling behaviour of the PEG/C/PVA hydrogel in salt solutions. At higher concentrations, increased electrostatic connections between the cationic and anionic parts of the polymer chain cause a rise in osmotic pressure and a decrease in the inflow of solution [60], due to which the swelling of the PEG/C/PVA hydrogel decreases. Anions in both electrolytes, such as chloride ions, are similar, whereas cations, such as monovalent sodium ions and divalent calcium ions, are different due to their different charge‐to‐size ratio [61]. So, swelling is further reduced in the case of CaCl_2,_ as it is difficult for larger ions to enter the network of PEG/C/PVA hydrogel [39, 62].

### 4.7 In Vitro Antimicrobial Analysis

**FIGURE 7:**
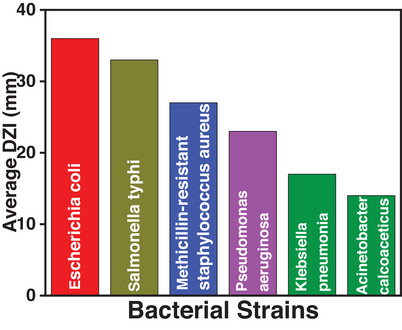
The antimicrobial activity of Ciprofloxacin loaded hydrogel, against pathogenic microorganisms.

The Kirby–Bauer Disc Diffusion method was used to investigate the antimicrobial activity of samples. The zones of inhibition were measured after 24 h of incubation. It is clear from Figure 7 that the PEG/C/PVA hydrogel did not inhibit the tested bacterial strain, as chitosan solubility is pH‐dependent. It is insoluble in neutral and alkaline solution [63], as shown in Figure S2. On the other hand, ciprofloxacin‐loaded PEG/C/PVA hydrogel and ciprofloxacin reference had antibacterial activity against the tested Gram‐positive and Gram‐negative bacterial strains. Both reference and ciprofloxacin‐loaded PEG/C/PVA hydrogel show inhibition zones of almost equal diameter. Ciprofloxacin‐loaded PEG/C/PVA hydrogel showed higher inhibition zones against *E. coli*, *S. typhi* and methicillin‐resistant *S. aureus*. It showed a smaller inhibition zone against *P. aeruginosa*, *A. calcoaceticus* and *K. pneumoniae* (Table 2). The data collected showed that the synthesized PEG/C/PVA hydrogel released the antibiotic drug (ciprofloxacin HCl) into bacterial cells, thereby inhibiting both Gram‐negative and Gram‐positive bacterial strains.

**TABLE 2:** The antimicrobial activity of ciprofloxacin‐loaded hydrogel, synthesized hydrogel, ciprofloxacin, chitosan hydrogel, PEG, PVP and TEOS against pathogenic microorganisms.

Sample	Gram negative bacteria					Gram positive bacteria
	*Escherichia coli*	*Salmonella typhi*	*Pseudomonas aeruginosa*	*Acinetobacter calcoaceticus*	*Klebsiella pneumoniae*	M.R.S.A
Ciprofloxacin‐loaded hydrogel	36	33	23	14	17	27
Synthesized hydrogel	NA	NA	NA	NA	NA	NA
Ciprofloxacin HCl	35	33	22	13	16	26
Chitosan hydrogel	NA	NA	NA	NA	NA	NA
PEG	NA	NA	NA	NA	NA	NA
PVP	NA	NA	NA	NA	NA	NA
TEOS	NA	NA	NA	NA	NA	NA

The PEG/C/PVA hydrogel, in its unloaded form, has no detectable antibacterial activity. This can be attributed to limited access to chitosan's free amino groups (─NH_2_) due to hydrogen bonding with PEG and PVA, as well as to crosslinking with TEOS during hydrogel network formation. This limits their ability to interact with bacterial membranes. In addition, because chitosan bears an insoluble structure and is less protonated, the antibacterial action of chitosan is dramatically reduced in neutral or slightly alkaline conditions [13, 64, 65, 66]. In contrast, the ciprofloxacin‐loaded hydrogel exhibited potent antibacterial activity, which can be ascribed to the controlled release of the antibiotic from the polymeric network under controlled conditions, indicating that the observed inhibition is mainly due to the released drug rather than the hydrogel matrix. Therefore, these results show that the prepared PEG/C/PVA hydrogel serves as an excellent carrier for sustained drug delivery rather than as an intrinsic antibacterial agent.

### 4.8 In Vitro Drug Release Study

The release of drugs from polymeric hydrogels occurs mainly through four mechanisms: Solvent‐controlled, degradation‐controlled, stimulus‐responsive and diffusion‐controlled.

In solvent‐controlled systems, solvent transport into the polymer network is crucial for drug diffusion. Such a mechanism can either originate from osmotic pressure or from swelling‐controlled release. Swelling‐controlled release is essential in hydrogels such as PEG/C/PVA, where water uptake increases the network mesh size and opens pathways for drug diffusion. The degree of swelling has a direct effect on the diffusion rate and the overall release of drug molecules [67, 68]. In the current study, the PEG/C/PVA hydrogel exhibited a swelling‐dependent release profile, indicating that solvent interactions drive drug transport.

Degradation‐controlled release relies on the cleavage of polymeric chains either by hydrolytic or enzymatic action, which leads to progressive matrix disintegration and prolonged drug release [69]. While this mechanism is common for biodegradable polymers, such as polylactic‐co‐glycolic acid (PLGA) or polycaprolactone (PCL), it was less relevant in the present case of PEG/C/PVA because the hydrogel retained its structure throughout the release timeframe [70]. Stimulus‐responsive release is defined as a release triggered by environmental factors, including pH, temperature or ionic strength [71]. Indeed, the present PEG/C/PVA hydrogel exhibited pH‐dependent swelling due to chitosan, whose charges are sensitive to protonation. However, the pH range considered herein (close to neutrality) kept the network bound via hydrogen bonds rather than allowing significant protonation.

The drug diffuses through the hydrated polymer matrix, driven by the concentration gradient in the diffusion‐controlled release [72]. Diffusion became the dominating release mechanism as the PEG/C/PVA hydrogel inflated. In vitro results (Figure 6d) indicated that ∼100% of diclofenac sodium and ciprofloxacin HCl were released in a controlled manner at approximately 180 and 150 min, respectively, and were compatible with the USP XXIV norm. The amino groups of chitosan and the nitrogen atoms of PVA were not protonated at pH 7.4, thereby allowing the hydrogel to remain crosslinked via hydrogen bonding and physical entanglement. These data confirmed that the PEG/C/PVA hydrogel is a potentially viable, stable and sustained drug‐release carrier.

### 4.9 Literature Comparison

The results obtained for drug delivery in this work are compared with the current biopolymer‐based systems, such as C/PVA/ECH, alginate/PVA, Na‐alginate, montmorillonite‐alginate, CTS‐g PAA/APT/SA and chitosan‐cyanocobalamin (shown in Table 3), which show that this study makes our PEG/C/PVA hydrogel more effective for drug delivery, because of its superior crosslinking, synergistic component combination and optimized network topology. The synthesized hydrogel released 100% of diclofenac Na within 180 min and 100% of ciprofloxacin HCl within 150 min, demonstrating fast yet controlled diffusion through its porous, hydrophilic matrix. TEOS was used as a crosslinker to enable mechanical stability and swelling capacity, effective water absorption without structural collapse. The combination of PEG, chitosan and PVA provides complementary qualities: PEG encourages diffusion and hydrophilicity, PVA increases mechanical strength and chitosan provides pH sensitivity and bioadhesion. When combined, these properties result in a hydrogel which is stable, economical and biocompatible, enabling complete drug release and outperforming many current natural and synthetic polymer systems.

**TABLE 3:** Comparison with literature.

Sr. No.	Hydrogel composition	Drugs	Percent release and time			References
			SGF (pH = 1.2)	SIF (pH = 6.8)	7.4 pH	
**1**	C/PVA/ECH	Diclofenac Na		94.51% in 130 min		[73]
**2**	Alginate/PVA			100% in 120 min		[74]
**3**	Na‐alginate		15% in 1–2 h		76% in 2–3h	[75]
**4**	Montmorillonite‐alginate		17.70% in 2 h	48.80% in 8h		[76]
**5**	CTS‐g PAA/APT/SA		3.76% in 2 h	100% in 24h		[77]
**6**	N‐trimethyl chitosan/sodium carboxymethyl xanthan gum	Ciprofloxacin HCl			96.1% in 150 min	[31]
**7**	Chitosan‐cyanocobalamin				75% in 600 min	[78]
**8**	**Present work**	Diclofenac Na		100% in 180 min		
		Ciprofloxacin HCl		100% in 150 min		

## 5 Conclusion

In the present study, the solution‐casting method was successfully used to synthesize a novel pH‐sensitive chitosan‐based PEG/C/PVA hydrogel for the controlled release of diclofenac sodium and ciprofloxacin HCl. The synthesized PEG/C/PVA hydrogel contains an intricate network of chitosan, PVP, PEG and TEOS. FTIR analysis confirms the presence of functional groups of polymers such as ─NH group of chitosan, inter‐ and intra‐molecular hydrogen bonding, acetamide group of chitosan, Si─N linkage, and Si─O─Si of TEOS. In contrast, SEM analysis reveals the smooth, porous surface of the PEG/C/PVA hydrogel. The maximum swelling was observed at acidic pH (pH 2) and at lower electrolyte concentrations (0.1 ppm). The gel content of the synthesized PEG/C/PVA hydrogel was 89.65%. The ciprofloxacin HCl‐loaded PEG/C/PVA hydrogel exhibited antibacterial activity against *E. coli*, *S. typhi*, methicillin‐resistant *S. aureus*, *P. aeruginosa*, *A. calcoaceticus* and *K. pneumoniae*. The in vitro release of ciprofloxacin HCl‐loaded PEG/C/PVA hydrogel demonstrated 100% release in 150 min, and diclofenac sodium‐loaded PEG/C/PVA hydrogel showed 100% release in 180 min in SIF (pH = 6.8). The drug release behaviour showed that synthesized PEG/C/PVA hydrogel is a potential candidate for the controlled release of diclofenac sodium and ciprofloxacin HCl.

## Funding

The authors have nothing to report.

## Conflicts of Interest

The authors declare no conflicts of interest.

## Supporting information




**Supporting File** ansa70065‐sup‐0001‐SuppMat.docx.

## Data Availability

The data that support the findings of this study are available from the corresponding author upon reasonable request.
