# Supplementary material for: Stimuli‐Responsive Crosslinked Chitosan/PVP/PEG Hydrogel Networks for Targeted Drug Delivery and Antimicrobial Performance
Source: Anal Sci Adv. 2026 Feb 10;7(1):e70065. doi: 10.1002/ansa.70065 (PMC12891981; doi:10.1002/ansa.70065)
Supplement: Supplementary file 1 — Supporting File ansa70065‐sup‐0001‐SuppMat.docx. [file ANSA-7-e70065-s001.docx]

**
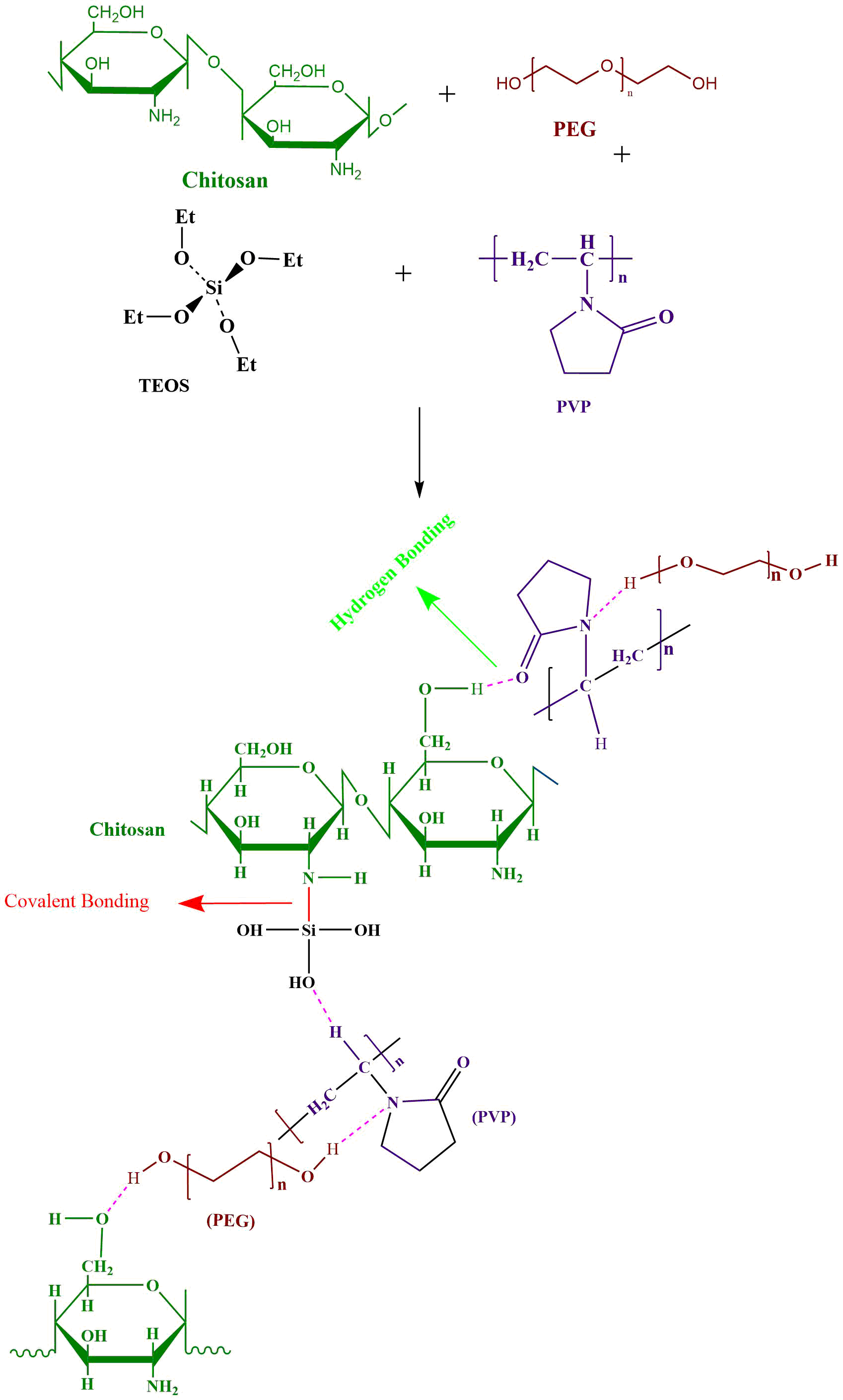
**

***S.1.*** *Proposed interaction between the constituents of the hydrogel*


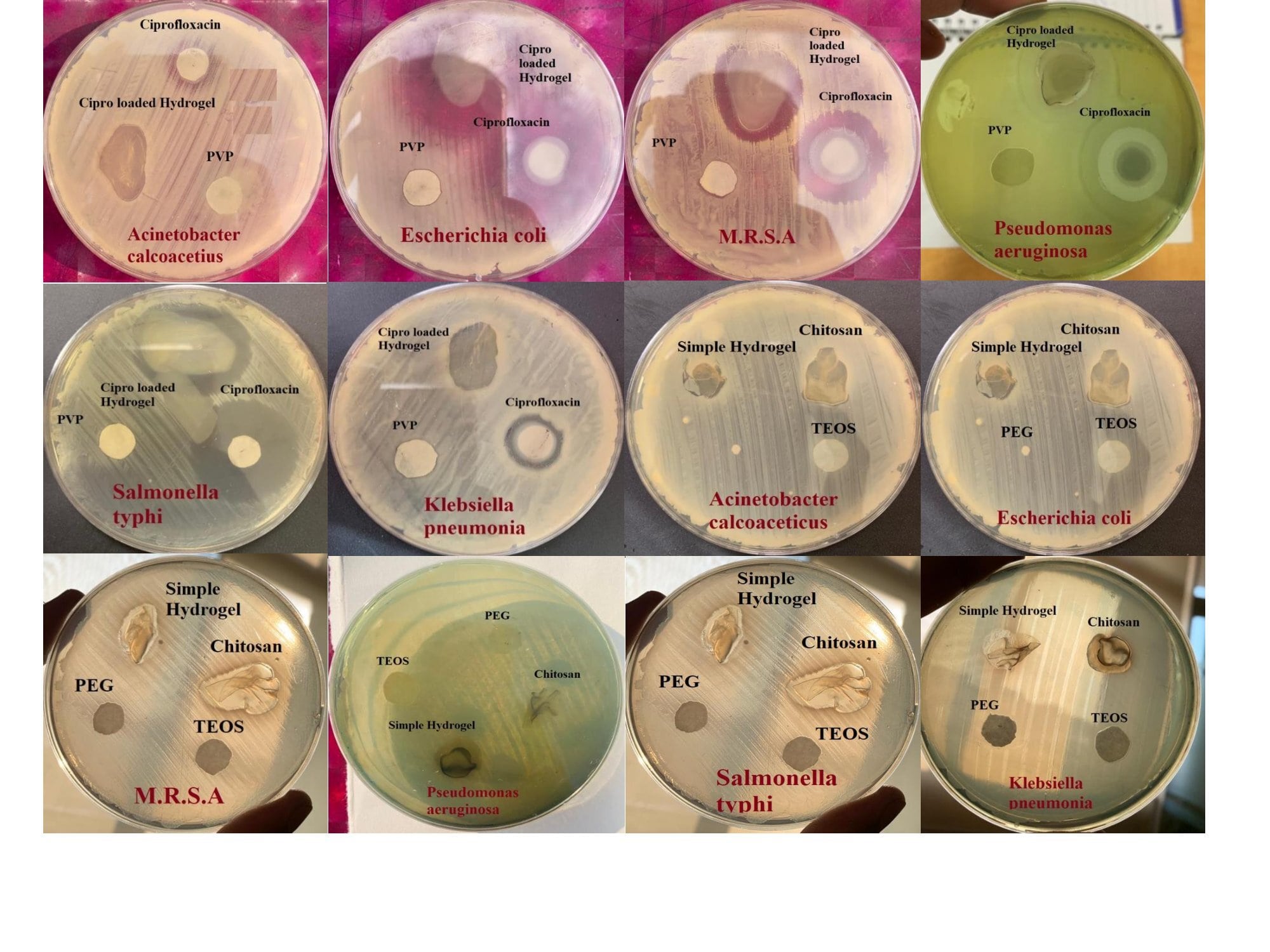


***S.2.*** *The antimicrobial activity of Ciprofloxacin loaded hydrogel against pathogenic microorganisms.*
